# Evaluation of an Online Campaign for Promoting Help-Seeking Attitudes for Depression Using a Facebook Advertisement: An Online Randomized Controlled Experiment

**DOI:** 10.2196/mental.3649

**Published:** 2015-03-18

**Authors:** Alison Hui, Paul Wai-Ching Wong, King-Wa Fu

**Affiliations:** ^1^ Journalism and Media Studies Centre The University of Hong Kong Hong Kong China (Hong Kong); ^2^ Department of Social Work and Social Administration The University of Hong Kong Hong Kong China (Hong Kong)

**Keywords:** depression, help-seeking, randomized controlled experiment

## Abstract

**Background:**

A depression-awareness campaign delivered through the Internet has been recommended as a public health approach that would enhance mental health literacy and encourage help-seeking attitudes. However, the outcomes of such a campaign remain understudied.

**Objective:**

The main aim of this study was to evaluate the effectiveness of an online depression awareness campaign, which was informed by the theory of planned behavior, to encourage help-seeking attitudes for depression and to enhance mental health literacy in Hong Kong. The second aim was to examine click-through behaviors by varying the affective facial expressions of people in the Facebook advertisements.

**Methods:**

Potential participants were recruited through Facebook advertisements, using either a happy or sad face illustration. Volunteer participants registered for the study by clicking on the advertisement and were invited to leave their personal email addresses to receive educational content about depression. The participants were randomly assigned into two groups (campaign or control), and over a four consecutive week period, received either the campaign material or official information developed by the Hospital Authority in Hong Kong. Pretests and posttests were conducted before and after the campaign to measure the differences in help-seeking attitudes and mental health literacy among the campaign and control groups.

**Results:**

Of the 199 participants that registered and completed the pretest, 116 (55 campaign and 62 control) completed the campaign and the posttest. At the posttest, we found no significant changes in help-seeking attitudes between the campaign and control groups, but the campaign group participants demonstrated a statistically significant improvement in mental health literacy (*P*=.031) and a higher willingness to access additional information (*P*<.001) than the control group. Moreover, the happy face Facebook advertisement attracted more click-throughs by users into the website than did the sad face advertisement (*P*=.03).

**Conclusions:**

The present study provides evidence that an online campaign can enhance people’s mental health literacy. It also demonstrates the practicality and effectiveness of an online depression awareness campaign using a Facebook-based recruitment strategy and distribution of educational materials through emails. It is important for future studies to take advantage of the popularity of online social media and conduct evaluative research on mental health promotion campaigns.

## Introduction

Mental illness is a substantial contributor to the global burden of disease [1]. However, mental illness remains underdiagnosed in many people, and many of them receive minimal to no treatment for their illnesses [2]. From the health policy perspective, one way to tackle the underdiagnosis problem is to deliver mental health campaigns for promoting acceptance, enhancing knowledge, and encouraging help-seeking behavior [3]. According to the World Health Organization, selective preventive intervention programs targeted at specific groups, such as school children, adolescents, and elderly persons can help reduce depression [4]. Some campaigns have been developed to address stigma or cultivate positive attitudes toward depression and its treatments [5,6], but their effects on encouraging help-seeking intention has yet to be measured [6].

Along the same line, some research findings suggest that an increase in mental health literacy can affect one’s help-seeking intention [7-9]. According to the findings of a systematic review of 22 studies [10], mental health literacy is listed as one of the important factors for strengthening help-seeking intentions for mental disorders among adolescents.

Health promotion campaigns delivered through the Internet have become increasingly more common recently [11,12]. The Internet is one of the major platforms for the public to seek health information and resources because of its convenience and anonymous nature [13]. Online campaigns, through the use of websites, online forums, and social media in particular, enable reaching diverse populations and providing motivation through reminders and feedback to the participants [14]. They can also mimic interpersonal communication to advocate health-related behavioral changes [15]. In a meta-analysis of 85 online intervention studies, online campaigns were found to be helpful for physical health behavior changes with statistically significant effects, especially when grounded in theory of behavioral change like the theory of planned behavior (TPB) [12,16].

Nevertheless, a systematic review revealed that there were very few effectiveness studies of online campaigns intended to encourage young people’s help-seeking for mental health, and indeed most demonstrated no statistically significant impacts [17]. The authors also found that most studies in the review were of low quality, suggesting further research was needed to evaluate the studies properly [17].

This study aims to evaluate the effectiveness of an online campaign targeted at enhancing help-seeking attitudes for depression in Hong Kong. Specifically, the study seeks to examine whether such a campaign can promote participants’ help-seeking attitudes for depression and mental health literacy. This study is the final stage of a multiple-phase, mixed-method research project aimed at developing a universal media campaign to encourage a help-seeking attitude for depression in Hong Kong. Hong Kong is a business-centric and fast-changing city with dynamic economic development and wide income gaps. These modern city characteristics have marked implications for the population’s mental health status [18]. However, very little research has been done to measure the prevalence of clinical mental disorders among the general public in the city [19]. Campaigns that promote awareness and anti-stigmatization for depression have arisen in the recent decades, but virtually no study has been rigorously conducted to evaluate the effectiveness of these campaigns in Hong Kong.

##  Methods

### Study Background

Theoretical models are needed to guide campaign development and strategies [12]. Formative research, including a series of semi-structural interviews, was conducted to formulate and validate a theoretical framework for help-seeking in depression in Hong Kong and the results have been reported elsewhere [20]. The research made reference to the TPB and the McGill Illness Narrative Interview (MINI) to allow theoretical, contextual, and cultural understandings of how various factors interweave and of the way in which an individual’s help-seeking attitude and behavior is influenced [20]. As a study outcome, a model was built with factors that explain how help-seeking intentions can be targeted for media campaigns. The model includes attitudes toward help-seeking, subjective norms, and perceived behavioral controls as suggested by the TPB, as well as the following three additional factors (1) attitude toward treatment, (2) perceived barriers and individual actions, and (3) perceived responses if a family member or friend experiences depression [21].

In addition, the quality of the campaign material was examined. One central question was how to design visual material for online display that can draw the largest extent of viewers’ attention to the material? Previous studies have demonstrated that people with depression have an attentional bias against negative and sad facial expressions [22,23]. As such, the second objective of this study is to examine click-through behaviors by varying the attractiveness of two affective photographs showing either sad or happy facial expressions in online advertisements.

The study protocols were approved by the Human Research Ethics Committee for Non-Clinical Faculties, The University of Hong Kong. Trial registration for this kind of health attitude research is not required. We followed the Checklist for Reporting Results of Internet E-Surveys (CHERRIES) for reporting the development and findings of online surveys [24].

###  Participants and Procedures

Participants who identified themselves as Hong Kong residents using traditional Chinese language and aged 18-59 years old in their Facebook profiles were recruited through paid advertisements on Facebook. Facebook has more than 845 million users around the world [25]. Facebook is an especially appealing medium to young adults [26-28] and is found to be particularly useful for follow-up research due to users’ frequent or daily usage [28]. Health-related research has successfully deployed online participant recruitments to reach out to larger audiences or specific demographics like young women or smokers, or for interventions in areas like HIV prevention [26,29,30]. As one of the most popular social networking websites, Facebook offers recruitment advertisements that can be arranged to be shown only to the targeted population through users’ age and location indicated on their profile or their Internet Protocol (IP) address [26,31]. Facebook identified and estimated a total of 1.3 million users as our target audience using the criteria of being a Hong Kong resident, 18-59 years of age, and Cantonese speaking.

With reference to the recruitment procedure of the previous studies, the cost-per-click advertising model was used [26,31]. This payment model goes along with a bidding system employing the bidding price range suggested by Facebook, which is the cost per click of the advertisements. Advertisements with lower prices for bids are less likely to be displayed. Also, limits can be set in order to control the cost for per day or for the entire campaign. When the limit is reached, Facebook stops showing the advertisement [26,31].

Two Facebook advertisements were posted during the period from January 29, 2013 to March 3, 2013, with more than 26 million prevalence of impressions representing the incidence of appearance on Facebook (ie, people may see multiple impressions of the same advertisement). A total of HK $17,887.87 (roughly US $2300) was spent on both advertisements that gained a total of 5405 clicks. The main advertisement copy was posted with the headline “Say no to depression” in Chinese. On the side, there was a short paragraph asking:

What would you do if you discovered that your friend might have depression? Click here and we will provide you with four episodes of electronic material related to depression for free.

**Figure 1:**
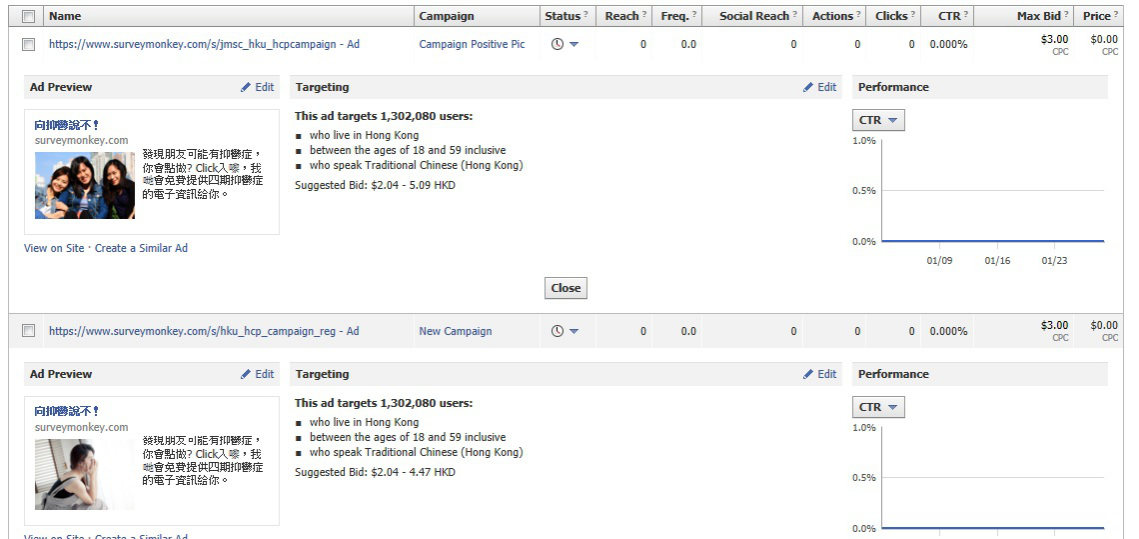
The happy and sad face images and their targeted Facebook users.

With the same copy, two versions of the advertisements were made with the following attached pictures (1) a depressed woman covering her face (sad face), and (2) three cheerful young ladies (happy face) (Figure 1).

**Figure 2:**
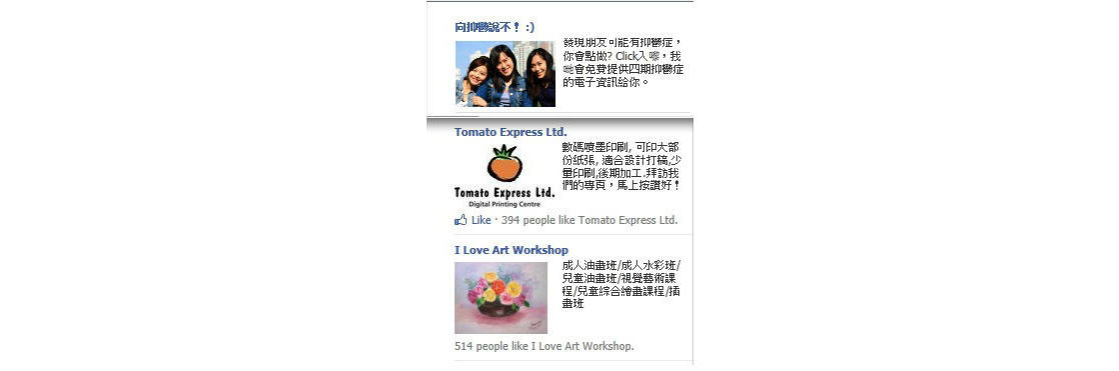
Example of the Facebook advertisement.

The Facebook advertisement (Figure 2) was linked to the introductory page of an online survey supported by SurveyMonkey [32]. The page provides explanation for the study purpose, introduces the flow of the campaign, and requests the user’s participation and consent to join the campaign. Participants can click the “Agree” button to give consent and then enter an email address for receiving future campaign material. We checked the email registration and the IP address collected by the system to avoid duplication of participants. Before the campaign, the user interface of the online survey was pilot tested and then modified to enhance usability.

The study’s internal validity was one of our concerns as participant recruitment and campaign execution were carried out completely on the Internet. In order to take into account possible duplications and to minimize the number of fake accounts or registrations, which are widespread concerns for online surveys and campaigns, participants were required to manually input the email registration after clicking into the Facebook advertisements. This email registration process and the following steps of sending questionnaires and campaign material through email helped minimize the chances of false identities and accounts. The IP addresses were also tracked to minimize duplication [33]. However, the process compromises the user convenience of simply doing a web-based survey where duplication and fake responses are inevitable. A pretest and posttest design was used to conduct the surveys. Drawing on the TPB’s three factors (1) attitude toward help-seeking, (2) subjective norm, and (3) perceived behavior control, the pretest and posttest were identical and aimed to measure the changes in the participant’s value and view of depression after the campaign. The items used as primary outcomes were derived from the help-seeking theoretical model as developed in the formative research stage. Secondary outcome measures included changes in mental health literacy [34], attitudes toward seeking professional help [35], and supplementary factors including action and view toward family and friends with depression, perceived barriers, and attitudes toward treatment. The Depression Anxiety Stress Scale (DASS) in Chinese was also used to measure the respondents’ depressive episode [36].

Using Cronbach’s alpha and confirmatory factor analysis, the reliability and validity in the measurements of the items were tested in a separate study that was reported elsewhere [37]. The Cronbach’s coefficients for attitude, subjective norm, and perceived behavior control were 0.77, 0.77, and 0.82, respectively. Confirmatory factor analysis showed an acceptable fit of the model.

The duration of the campaign was six weeks. Participant registration, which was also the end of the posting period for the Facebook advertisement, was due on March 3, 2013. The web link of the pretest was then sent out for all registered participants’ email accounts. The cut-off date for completion of the pretest was March 18, 2013, with prompt reminders sent beforehand. Those who failed to fill in the pretest were considered as dropouts from the campaign.

Every Monday for the following four weeks, a short paragraph of campaign material on depression was sent out in email format to participants. Participants had been randomly assigned to two groups that received either originally developed material for this campaign or the official mental health material prepared by the Hong Kong Hospital Authority, which was used as a control comparison. Participants were not aware of which group they belonged to. The full set of online questionnaires is provided (Multimedia Appendix 1).

###  Campaign Material

The campaign material was originally designed by the first author, who has a bachelor’s degree in journalism and has received mental health first-aid training, and was reviewed by the second author, who is a clinical psychologist and mental health researcher. All campaign material was written in Chinese. Four episodes of campaign messages were created with reference to the factors in the help-seeking theoretical model with each week featuring a background of depression, attitudes toward help-seeking, subjective norms, and perceived behavioral control [21]. The four email scripts were based on a story that was developed originally to incorporate the above factors. The story depicts a sister who suspects her brother has depression, and in turn portrays the progression of events as the sister and brother seek information and care (Multimedia Appendix 2).

An external web link was printed at the end of the email body for optional further reading, which provided additional information related to the week’s message. The additional information was adopted from existing health information sourced from the World Health Organization, Hong Kong Hospital Authority, and academic studies related to depression [38,39] and posted on the online survey page with a normal web page layout. In order to monitor who clicks into the further readings, the page starts with a question that asks whether the participant has read any other depression-related information during the week. The next page has the actual further reading information and ends with a question asking whether the information was helpful. The two-step process of answering questions and clicking “Next” and “Done” helped measure which participants clicked into the halfway point and which ones completed the further reading.

On the Monday of the sixth week, a link to the posttest was sent out in email format to all participants. In the final email, participants were advised to fill in the posttest only after they had finished reading the previous four weeks’ material. Participants were given a week to fill in the posttest. The posttest was due at the end of week 6. Both pretest and posttest were voluntary surveys without incentive. Participants who did not complete the questionnaires or did not fill in the required material were considered to be dropout participants.

The chi-square test was used to test the differences between campaign and control groups for the variables described in Table 1. The Fisher's exact test was used if any cell frequency was fewer than five. The t test was deployed to test the mean difference between groups. The campaign outcomes were assessed by the analysis of variance (ANOVA) model in the statistical software package SPSS 21.0. P value was set as .05.

##  Results

**Figure 3:**
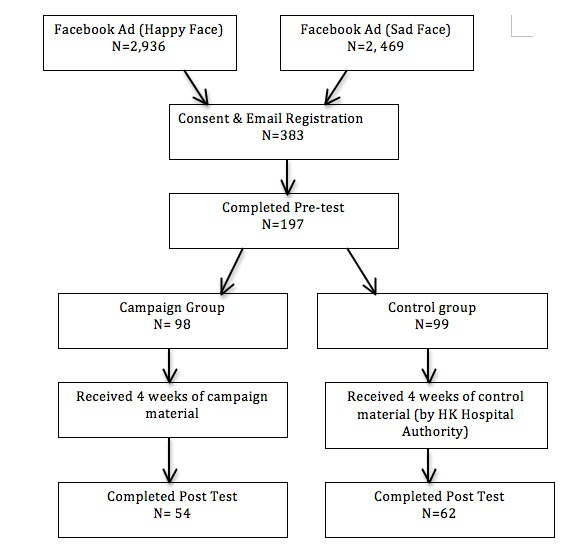
Flow chart of the study.

Of the 383 Facebook users who had consented and registered with their personal email address to join the campaign, 197 (51/100, 51.0%) completed the pretest and were included in the campaign. They were randomly assigned into one of the two groups with 98 in the campaign group and 99 in the control group. After receiving four weeks of electronic campaign material, of the 116 participants that completed the posttest, 54 (55/100, 55.0%) were in the campaign and 62 (63/100, 63.0%) in the control group. The participants’ flow of this study is shown in Figure 3. 

No significant difference in demographic characteristics was found between the two groups (Table 1).

Participants were mainly young adults, 18-29 years old, who represent the most frequent Internet and social media users. Over 50% of them had attained the secondary school education level. Also, about 30% of the participants reported that they had received a diagnosis of depression before. More than 70% of the participants reported suffering from mild to extremely severe depressive symptoms.

**Table 1:** Demographic characteristics of participants of the campaign and control groups.

Demographic characteristics		Campaign (N=55)	Control (N=62)	Difference (%)	*P* values
		n (%)	n (%)		
**Gender**					0.13
	Male	23 (43)	17 (27)	16	
	Female	31 (57)	45 (73)	-16	
**Age**					0.84
	18-29	24 (44)	22 (35)	9	
	30-39	6 (11)	8 (13)	-2	
	40-49	10 (19)	11 (18)	1	
	50-59	12 (22)	19 (31)	-9	
	60 or above	2 (4)	2 (3)	1	
**Education level**					0.35
	Primary school	0 (0)	4 (6)	-6	
	Secondary school	30 (56)	32 (52)	4	
	Undergraduate	19 (35)	21 (34)	1	
	Postgraduate	5 (9)	5 (8)	1	
**Ever been diagnosed with depression?**					0.26
	Yes	13 (24)	22 (35)	-11	
	No	41 (76)	40 (65)	11	
**Ever sought professional help?**					0.70
	Yes	25 (46)	32 (52)	-6	
	No	29 (54)	30 (48)	6	
**Friends or family members who had a diagnosis of having depression?**					0.88
	Yes	33 (61)	36 (58)	3	
	No	21 (39)	26 (42)	-3	
**Depression anxiety stress scale (DASS) level**					0.36
	Normal	13 (24)	15 (24)	0	
	Mild	3 (6)	6 (10)	-4	
	Moderate	12 (22)	16 (26)	-4	
	Severe	10 (19)	4 (6)	13	
	Extremely severe	16 (30)	21 (34)	-4	

While comparing the click-through rate to the optional further readings between groups, 69 participants from the campaign group (70%, 70/100) clicked through at least once during the four weeks, whereas only 40 participants from the control group (40%, 40/100) clicked through, suggesting that the participants in the campaign group were more willing to read further information than those of the control group (χ^2^_1_=16.7, *P*<.001).

The primary and secondary outcome measures in the help-seeking model are presented in Table 2. The analysis showed that changes in all primary outcomes including attitude, subjective norm, perceived behavioral control, and intention for help-seeking of depression, which were adapted from the core variables of the TPB, were found to be statistically insignificant. However, significant changes were found in mental health literacy between the two groups. In other words, the campaign group participants were more likely to recognize that the character portrayed in the vignette was experiencing depression than did respondents in the control group. In the campaign group, the percentage of participants who were able to recognize depressive symptoms in the vignette increased from 40%-68%, whereas a decrease was found in the control group from 59.7%-53 % (χ^2^_1_=4.5, *P*=.034).

**Table 2:** Posttest scores of the primary and secondary outcome measures of the help-seeking models in the campaign and control groups.

Outcome measure		Campaign (N=54)	Control (N=62)
		Mean (SD)	Mean (SD)
**Primary**			
	Attitude	^a^2.05 (0.46)	2.07 (0.55)
	Subjective norm	2.84 (1.46)	2.56 (1.61)
	Perceived behavioral control	2.49 (1.31)	2.29 (1.33)
	Intention	3.01 (1.48)	2.73 (1.58)
**Secondary**			
	Perceived barrier	4.35 (1.48)	4.26 (1.69)
	Attitude toward treatment	2.58 (1.11)	2.47 (1.21)
	Action and view toward family and friends with depression	3.40 (1.10)	2.73 (1.58)

^a^1 is the highest likelihood to seek help, and 7 is the lowest

We also tested which picture, happy- or sad-looking, received more clicks to join the campaign. The advertisement with the happy face gained 2936 clicks with 12,591,361 impressions, representing a 0.023% (0.023/100) click-through rate into the advertisement and HK $3.05 (US $0.39) per click. The sad face advertisement gained 2469 clicks out of 13,412,509 impressions, amounting to a 0.018% (0.018/100) click-through rate and HK $3.62 (US $0.46) per click. The total spending of the happy and sad face advertisements was HK $8,942.89 (US $1,146.50) and HK $8,944.98 (US $1,146.80), respectively. The click-through rate of the happy face picture was statistically significantly higher than the one of the sad face (χ^2^_1_ = 75.1, *P*<.001). Among those completing the pretest, no significant difference in demographics was found between those who clicked into the two advertisements. However, there were significantly more pretest completers who had a diagnosis of depression among those who clicked into the happy face advertisement than those who clicked into the sad face (chi-square test with Yates' continuity correction, *P*=.03).

##  Discussion

### Principal Findings

This study demonstrated the possibility of delivering an online mental health campaign that promotes help-seeking for depression. Although no significant change was detected in the primary outcomes of the campaign, which were designed to closely following the TPB, the increase of mental health literacy in the intervention group provides supportive evidence in the educational value of such campaigns. The result is consistent with a similar campaign evaluation study that found that social media campaigns can improve mental health literacy but have limited impact on attitudinal outcomes [40]. Previous research has also supported the effectiveness of improving mental health literacy in public campaigns as it could help encourage early identification and intervention for mental disorders [41,42].

It is important to note that our campaign was particularity attractive to individuals with mild to severe depressive symptoms, irrespective of who were or were not receiving treatment. We found that 30% (30/100) of the participants had a diagnosis of depression, and more than 70% of participants reported that they were mildly or severely depressed. This might reflect that participants who were suffering from depressive episodes were particularly attracted to the depression-related advertisement on Facebook and campaign material. Since nearly half of the participants have sought professional help for their depressive episodes, their help-seeking intention might have been shaped by their own personal illnesses and treatment experiences. This may partly explain the non-significant changes in the primary outcomes of the help-seeking intention but rather significant improvement in media literacy [43,44]. Hence, we argue that the insignificance of some of the results of this study cannot reject the importance of this theory-based campaign and the usefulness of theories like TPB, but further research is needed. Indeed, some participants left messages at the end of the campaign noting that they had experienced or were experiencing depression and hoped that their participation in the study would be helpful for research on depression and help-seeking.

Another interesting finding concerns the different responses by those receiving the two pictures in the Facebook advertisement. The Facebook advertisement with a happy face picture gained more clicks than did the one with a sad face. According to our analysis, no significant difference in demographics was found between those who had clicked through the two advertisements. However, we found more people who had a diagnosis of depression among those who clicked into the happy face advertisement than those who clicked into the sad face, which runs contrary to previous laboratory findings of depressive patient’s differential attention to emotional pattern [22,23]. This finding has practical implications for informing campaign developers in that they should pay attention to content design and use appropriate material for the campaign, especially when the campaign is customized for those people with a history of mental health problems.

Facebook is increasingly being used for participant recruitment for research, especially targeting specific groups like adolescents, groups with specific health needs, patients, and caregivers [26,45]. Psychological research has also started to move online to explore more potential benefits of online research including lowering the cost of collecting data [3,25,45], conducting large scale observational [46], and experimental studies [47]. Research finds that Facebook is also very helpful in accessing the hard-to-reach populations due to its massive number of users worldwide and by recruiting participants through snowball sampling [48]. As our study topic , mental illness, is a socially stigmatized subject, we can specifically choose the target audience we want our advertisements to reach (ie, Chinese-speaking Hong Kong adult residents). It is also convenient for the participant to be recruited, and participate in the online campaign and surveys at their convenience and in their own space, as long as they have access to the Internet via phone or computer [45,49]. Since an individual’s online presence can be impersonal, research has found that it is sometimes easier for sensitive topics to be researched and communicated through the Internet as participants can remain anonymous when accessing the health information resources or participating in the campaigns [25,45,50]. Recent research has found that Facebook is a favorable health resource platform for groups with specific health needs to communicate, share information on the health problem and treatment experiences, provide emotional support, and communicate with medical workers [45,50]. Moreover, there is a lot of potential for analyzing Facebook or other social media platforms for big data health research [51].

### Limitations

The following are limitations worth noting. The recruited subjects may be biased toward certain characteristics (ie, relatively more female respondents and depressed patients). The dropout rate of the campaign can be considered high, which is not uncommon in online subject recruitment. The results might not represent those who dropped out from the email registration and the pretest. Due to the complications of reading campaign material and receiving pretest and posttest data through email, the dropout rate was higher than expected, but duplicated and fake responses were minimized since each registration and all email content was accomplished by a unique link and email message, which can ensure good data quality.

The loss of samples resulted in lower statistical power and might partly account for some of the non-significant statistical tests. In an online setting, there is no simple way to ensure that the subject has read and fully comprehended the material. But this factor should not alter the final results because both intervention and control groups should have the same impact, if any. Although it is acknowledged that help-seeking intentions may not directly lead to actual help-seeking behavior, effective preventive measures are essential in response to the prevalent reluctance regarding help-seeking for depression [52].

With the small sample size of our study, the conclusions may not be optimally representative to the public’s view of help-seeking and depression. However, using a theory-driven and culturally-specific campaign design strategy is recommended because it enables systematic understanding of public knowledge and allows room for the development of audience-oriented material. Although the change in help-seeking intentions was not significant in the current study, future studies can build upon our findings to replicate the study, revise the model, or carry out tests with a larger and more representative sample size.

###  Conclusions

Under the backdrop that clinical depression is projected to become a major global disease burden in the future, encouraging help-seeking for depression should be an essential aspect for health service development. Online media can help reach large numbers of participants and get help-seeking messages across to a population. In this study, an online media campaign, with the support of a theory-driven approach and customized campaign material, was used as an innovative and practical platform for a mental health campaign to encourage help-seeking for depression and to increase the individual’s media literacy. Further research is needed to confirm the results.

## Appendix

Multimedia Appendix 1 Full set of online questionnaires.

Multimedia Appendix 2 Campaign Material (Originally in Chinese).

## Abbreviations

DASS: depression anxiety stress scale
IP: Internet Protocol
TPB: theory of planned behavior
