# Supplementary material for: Evaluation of an Online Campaign for Promoting Help-Seeking Attitudes for Depression Using a Facebook Advertisement: An Online Randomized Controlled Experiment
Source: JMIR Ment Health. 2015 Mar 18;2(1):e5. doi: 10.2196/mental.3649 (PMC4607380; doi:10.2196/mental.3649)
Supplement: Multimedia Appendix 1 [file mental_v2i1e5_app1.pdf]

# Online Survey

## Pre-test Only

**Gender:**
Male
Female

**Age:**
18-29
30-39
40-49
50-59
Above 60

**Education Level**
Primary School
Secondary School
Undergraduate
Postgraduate

**Have you been diagnosed with depression?**
Yes
No

**Have you ever sough professional help?**
Yes
No

**Do you know of any friends or relatives who has been diagnosed with depression?**
Yes
No

**DASS(21)**
1) I couldn't seem to experience any positive feeling at all.

2) I find it difficult to work up the initiative to things.

3) I felt that I have nothing to look forward to.

4) I felt down-hearted and blue.

5) I was unable to become enthusiastic about anything.

6) I felt I wasn't worth much as a person.

7) I felt like life was meaningless.

**<u>Pre-test and Post-test</u>**

**<u>Mental health literacy</u>**

John is a 30-year-old who has been feeling unusually sad and miserable for the last few weeks. He is tired all the time and has trouble sleeping at night. John doesn't feel like eating and has lost weight. He can't keep his mind on his work. He puts off making any decisions and even day-to-day tasks seem too much for him. His boss is very concerned about the changes in his work performance.

1) What do you think is wrong with John?

2) What do you think can be helpful for John?

**<u>TPB Factors:</u>**

**<u>Attitude (Item 1-2: Subjective Norm, Item 3-6: Perceived Behavioral Control)</u>**

If I have depression, I think….

1) My family and friends will support me to seek help
2) My family and friends would not think seeking help is useful.
3) I can decide to seek help or not
4) To seek help or not is my personal choice
5) I can seek help

6) I can seek help if I want to

**Perceived view of family and friends with depression**

If my family or friend is experiencing depression, I think…

1) I can easily notice it
2) I will discuss with him about his emotional status
3) I will encourage him to seek help
4) He will listen to my advice to seek help
5) Discussing with him about his emotional status is easy to do
6) Convincing him to seek help is easy to do

**Treatment (Item 1-6: Attitude, Item 7-10: Intention)**

If I have depression, I think…'

1) Seeing psychiatrist is helpful
2) Seeing counsellor is helpful
3) Seeing psychologist is helpful
4) Medication is helpful
5) Professional help is affordable
6) Professional help is accessible
7) I prefer to seek help
8) I will try to seek help
9) I plan to seek help
10) I will seek help even if no one supports me

**Attitudes Toward Seeking Professional Help Assessment**

1. If I believed I was having a mental breakdown, my first inclination would be to get professional attention.

2. The idea of talking about problems with a psychologist strikes me as a poor way to get rid of emotional conflicts.

3. If I were experiencing a serious emotional crisis at this point in my life, I would be confident that I could find relief in psychotherapy.

4. There is something admirable in the attitude of a person who is willing to cope with his or her conflicts and fears without resorting to professional help.

5. I would want to get psychological help if I were worried or upset for a long period of time.

6. I might want to have psychological counseling in the future.

7. A person with an emotional problem is not likely to solve it alone; he or she is likely to solve it with professional help.

8. Considering the time and expense involved in psychotherapy, it would have doubtful value for a person like me.

9. A person should work out his or her own problems; getting psychological counseling would be a last resort.

10. Personal and emotional troubles, like many things, tend to work out by themselves
