# Supplementary material for: Evaluation of an Online Campaign for Promoting Help-Seeking Attitudes for Depression Using a Facebook Advertisement: An Online Randomized Controlled Experiment
Source: JMIR Ment Health. 2015 Mar 18;2(1):e5. doi: 10.2196/mental.3649 (PMC4607380; doi:10.2196/mental.3649)
Supplement: Multimedia Appendix 2 [file mental_v2i1e5_app2.pdf]

APPENDIX B

Campaign Material (Originally in Chinese)

Week 1:

Hi, this is Sze Sze. I want to share with you about my elder brother's story.

My brother is Ah Man. We have been really close since we were kids. Up till now we are still really close even we are both working grown-ups. However, I have observed that my brother has become very unhappy. He has been very tired all day. He did not have a good appetite and had very bad sleeping quality. He has lost interest in his usual hobby and did not want to meet with his friends. He could not concentrate at work and have lost incentive in doing everything. He knew that he isn't doing well but he did not know what to do.

I realized the changes in my brother and feel very worries but did not know what to do so I just initiated to chat with him. At first, he didn't want to talk much but I forced him for a few times and he was finally willing to chat. In our conversation, I found out that my brother seem to be experiencing the depressive symptoms I saw from the leaflets. I initiated to look for depression related information with him to see if he might be experiencing depression. In order to help family and friends with depression, we have to first know about depression…..

Week 2:

Remember I share last week about my brother having depression? We ended up read a lot of material related to depression and suspect that my brother might really be having depression. We think we should seek professional help and receive suitable treatment so we started looking for information on professional help.

I used my personal network and asked for advice from different friends. I have a few friends who had depression and had sought help so they gave me the contact of several psychiatrists and clinical psychologists. They also advised that I can first find help from family doctor and social worker.

Some of my friends had sought helped from psychiatrists and had received medication and psychological treatment from clinical psychologist at the same time. They shared that we should not face emotional issues alone because professional help is very helpful in relieving depression. In the process of seeking professional help, they can talk about their own issues and also receive professional treatment, which was helpful in solving emotional issues. Therefore, I have discussed with my brother and see which treatment he prefers and plan to accompany him to seek help.

Week 3:

Remember last week I told you about how my brother and I had searched for different treatment for depression? We realized that seeking help is not as difficult as we thought. My brother studied the material for professional help-seeking and decided to seek help. He realized he was able to receive different professional help and he can choose the most suitable treatment according to his own preference. If you or your family would experience depression, you will have the same ability to seek these professional helps. Your depressive symptoms can also be relieved.

Week 4: Following what I shared in the past few weeks, my brother took my advice and companion and finally received treatments that were suitable for him, which he found great relief. My brother recalled that if I didn't offer to help and support him, it might have taken him lots of effort to seek professional help.

My brother did not know if he was experiencing depression at that time. He said he was thankful that I realized his situation and encouraged him and went with him to seek help instead of blaming him or condemning him. Although we both do not know much about depression, he thinks that the determination of us facing it together has brought him hope and understanding that depression is nothing fearful.

My brother encourages everyone that we could share carefreely with our family and friends if we were to experience unhappy incidents and depressive episodes. Your family and friends will surely accept you and walk you through!
